# Knowledge, attitudes and practices regarding gemstone therapeutics in a selected adult population in Pakistan

**DOI:** 10.1186/1472-6882-9-32

**Published:** 2009-08-26

**Authors:** Sidra Ishaque, Taimur Saleem, Waris Qidwai

**Affiliations:** 1 Medical College, The Aga Khan University, Stadium Road, Karachi 74800, Pakistan; 2 Department of Family Medicine, The Aga Khan University, Stadium Road, Karachi 74800, Pakistan

## Abstract

**Background:**

Gemstones have been in use as part of alternative and complementary medicine for years. However, our understanding of the perceived healing powers of gemstones is limited. An extensive literature search revealed that there is a dearth of validated information on this subject. This study was therefore undertaken to explore the various aspects of the knowledge, attitudes, and practices of the public towards gemstone therapeutics.

**Methods:**

A survey was performed in the Community Health Centre of a tertiary care teaching hospital in Pakistan. Data collection was done via a face-to-face interview based on a structured, pre-tested questionnaire. Participants included all willing persons between 18–75 years of age approached prior to their appointments at the Community Health Centre.

**Results:**

The survey response rate was 86% (400/465). More than half (63%) of the study population was aware of the use of gemstone therapy. One hundred fifty-six individuals believed that gemstone use impacts health. Of this group, 39% believed that gemstone use increases physical strength. 62% believed that gemstone use is based on superstitious beliefs, whereas 28% opined that it is based on religious beliefs. 38% had used gemstones therapeutics formerly, while 24% were current users. Multiple logistic regression analysis revealed that age status and education status were significant (p < 0.05) independent predictors for both awareness of gemstone therapy and the belief that gemstone use impacts health. The elderly (aged 51–61) were 5.9-times more likely to believe that gemstones had an impact on health than the younger population (aged 18–28 years). (Adjusted Odd's Ratio = 5.9 [95% Confidence Interval = 2.9–11.9]).

**Conclusion:**

More than half of our sample population is aware of the use of the gemstones for their various effects. Willingness to use gemstones is associated with the beliefs about the impact of gemstone therapy on health. Friends and family seem to be the major role players influencing people's willingness to use gemstones. CAM modalities should be recognized and considered as an important therapeutic option. We feel that gemstone therapy is a relatively unexplored area and more studies should, therefore, be conducted to gather more validated information on the subject.

## Background

A gemstone is any mineral or petrified substance of beauty and durability that can be cut and polished for setting into a piece of jewelry or used for human adornment in other ways [1,2]. Gemstone therapy is most aptly described as a holistic and non-invasive therapy that involves wearing precious and semi-precious stones to improve physical and emotional health [3,4].

Gemstones therapeutics is a part of complementary and alternative medicine (CAM). CAM refers to a group of medical and health systems, practices, disciplines and products that are not considered a part of conventional medicine [5,6]. According to estimates, more than 80% of the developing world utilizes CAM, and half of the developed world also uses CAM [7]. The 'raison d'etre' for using CAM includes dissatisfaction with conventional medicine [8] via failed treatments and high costs as well as high expense incurred from conventional medicinal treatments [9]. However, this does not imply that these are the only reasons why people would opt for CAM. Other reasons that have been cited for choosing CAM include more active role of the patient in health maintenance, emphasis of CAM on treating each individual as a whole, faulty patient-doctor communication and lack of a conventional treatment for the disease [8,10]. Moreover, utilization of CAM has been found to be predicted by anxiety, back problems, chronic pain and urinary tract dysfunction. Dissatisfaction with conventional medicine did not predict CAM use, nor did sex, age, income or race/ethnicity [11]. Finally, people turn to CAM because they believe mind/body therapies are important for health maintenance or simply because they find it an interesting concept worth experimentation [12].

Apart from gemstone therapy, medicine homeopathy, acupuncture, chiropractic, flower essences and radionics are also included in alternative medicine. Patients with inflammatory bowel disease, arthritis and mixed chronic conditions have used CAM, as was observed in a study that examined the patterns of provider-based CAM use across three chronic illness groups [13]. In addition, infections, constipation, diarrhea, pregnancy related issues [8], gynecological problems [14] and kidney stones have all been treated under the umbrella of CAM. Aquamarine water has been used to treat conditions such as arthritis and scleroderma [15].

An exhaustive literature search for gemstone therapy revealed a large body of unvalidated information on the internet. The search for published and validated materials on the subject in peer reviewed medical journals was, however, not as fruitful. No large clinical studies have been carried out on this subject to date. During our literature search, we found only one study that was done to evaluate the effects of Hessonite on MDA MB 231 human breast cancer cells. The study suggested an inhibitory effect of Hessonite on these cancer cells [16].

Gemstone therapy's long history is documented in ancient texts. For example, in ancient Vedic texts from India, the origin and healing powers of various gems are described [3]. Similarly, Unani (Greek) texts detail disease treatments with pulverized gemstones [17].

Gemstone therapy is most commonly practiced as either electronic gem therapy or colour/radiation gem therapy. Notably, these are the only therapies for which some evidence is available regarding their application in alternative medicine. Color/stone radiation therapy uses an accelerated process of light projection and is believed to aid in the acceleration of healing processes [18] while electronic gem therapy uses dielectric resonating properties of gemstones to induce energy into diseased or injured tissues [19].

Believers in gemstone therapy feel that all gemstones carry certain vibrations and channel good energy. When the gem or stone is placed within a person's "aura", it can change the mental and physical outlook of the wearer. [4] "Crystal Healing" is also believed to help absorb, diffuse and focus energy into the body [13]. "Chakra" therapies involve a gemstone being placed on major and minor chakra points of the body to restore balance and vitality to the human system [20].

Gemstones are believed to be helpful in a number of disease states. Renal and gallstones have been particularly traditionally treated through gemstone therapy; this may be because of the correspondence in the state of matter between renal stones, gallstones and gemstones. In addition to enhancing physical and psychological well being, gemstones are purported to influence fortunes and finances. Some believe they evoke emotions, virtues and sexuality [3,4,20]. Proponents of gemstone therapy also advocate it as a much healthier and natural alternative to healing than taking prescription medication [4]. Although not dismissing these claims as superstitious, we should recognize that they largely emerge from anecdotal and traditional beliefs – sources of knowledge that are different from peer reviewed medical literature.

Pakistan currently has a pluralistic health care system where a number of components are involved in the delivery of heath care to the public. Financial constraints are an important consideration in the choice of the health care system [21]. We believe that gemstone therapy is a popular modality of therapy amongst the Pakistani population; though we have no baseline data on the subject. Our opinion and observation is based on patient accounts in clinical encounters and discussion with colleagues on gemstone therapy.

An integration of alternative and conventional medicine can go a long way in improving the health status of the population and subsequently improving their quality of life [22]. But for this purpose, treatments offered by alternative medicine need to be scientifically evaluated to make them evidence based. Our understanding of the perceived healing powers of gemstones is still incomplete. As a first step, we need to explore the knowledge, attitudes and practices of users of gemstone therapeutics to see whether it warrants scientific evaluation based on prevalence alone. Experiments could then be planned to study effects of gemstone therapy [3].

## Methods

### Study Design and Study Site

We conducted a knowledge, attitude and practices (KAP) survey at the Community Health Centre (CHC) at The Aga Khan University Hospital (AKUH), Karachi, Pakistan. AKUH is a tertiary level teaching facility in the private sector and caters to the medical needs of a large majority of patients coming from all over Karachi. The waiting area of the CHC clinics was chosen as the study setting because it is attended by a large number of people from all walks of life daily and hence caters to patients from almost all socioeconomic classes. The CHC not only runs family medicine clinics, but also provides specialist services to a multitude of patients.

### Study Sample and Data Collection

We required a sample size of 420 to fulfill our objectives at a 95% confidence interval, and 5% sample error. This sample size was calculated assuming a 50% variance and adjusting for a 10% non-response rate. Convenience sampling was used to draw the sample. All consenting individuals, visiting the CHC between 11 am to 6 pm and falling in the age bracket of completed 18 to completed 75 years of age were interviewed. Information was collected using face-to-face interviews based on a structured, pre-tested questionnaire. Informed consent for participation in the survey was obtained from the respondents of the survey. Strict confidentiality was maintained throughout the process of data collection, entry and analysis. The time period for data collection was two months (January 2008 to March 2008). The questionnaire was thoroughly discussed among the interviewers before data collection to decrease interview bias.

## Questionnaire

### a. Development

The initial questionnaire was developed based on the prior experience of investigators, input from colleagues, peers as well as patients. The initial framework of the questionnaire was then expanded by incorporation of new aspects encountered during an extensive literature search. The draft so prepared was then pre-tested on 25 respondents and no changes were deemed necessary to be made in the questionnaire based on this pre-testing. The results of the pre-testing were not included in the final analysis of the data. A meeting of the investigators was held prior to the administration of the questionnaire in order to maintain uniformity in its administration; hence reducing chances of interviewer's bias in the study.

### b. Sections

The questionnaire was divided into four sections. [see Additional file 1]

#### Section 1

The first section comprised of socio-demographic information. Education status was categorized into different groups ranging from illiterate (being the most uneducated) to post graduate (being the highest level of education). Therefore, "education upto class 12" is the category that includes people who had education only upto grade 12 but not beyond. Other categories were similarly delineated.

#### Section 2

Section 2 assessed the knowledge of respondents regarding gemstone therapy. This was assessed via questions related to the use of gemstones for their affects on health, identification of areas of health where gemstones are used like treatment of gall stones, renal stones or other(s) that the respondents might know of, methods of use of gemstones and source(s) of information regarding gemstone therapeutics.

#### Section 3

Section 3 comprised of questions assessing the attitudes of respondents regarding therapeutic use of gemstones. These questions related to opinions regarding beliefs about the impact of gemstones on health, finance, physical strength, longevity or other factor(s), whether the use of gemstones for health improvement is a superstitious or religious belief.

#### Section 4

Section 4 comprised of questions assessing the practices of respondents regarding therapeutic use of gemstones Questions dealing with areas such as advice and use of gemstones for therapeutic purposes were included.

### Statistical Analysis

Data was entered and validated using Epi-data version 3.1. It was cleaned for invalid and out of range values, missing values and duplicate ID numbers. The data was then imported to Windows Statistical Package for Social Sciences (SPSS) version 15.0 using DBMS. The data was analyzed using Windows SPSS version 15.0. As part of descriptive statistics, mean, median and mode were used to express the data collected. Associations were assessed using Chi-square test. P values were determined; a P-value < 0.05 was considered significant. Sociodemographic variables with a significant p value in chi square testing were subjected to a multiple logistic regression analysis to determine which factors were independent predictors of awareness of use of gemstones and the belief that gemstones impact health. All odds ratios were recorded with a 95% confidence interval. Tables and figures were used for an all-inclusive viewing of the results.

### Ethical Considerations

All efforts were made in this study to fulfill the ethical considerations in accordance with the Delcaration of Helsinki [23]. The confidentiality of each participant was strictly ensured throughout the project. The study was approved by the Ethical Review Committee at the Department of Community Health Sciences Department, Aga Khan University Hospital.

## Results

### Descriptive Statistics

#### Socio-demographic Characteristics of Study Population

We approached a total of 465 individuals for our survey, 45 of them declined to participate in the study. Of the 420 individuals who agreed to participate in the survey, 400 completed the interview. The response rate of the study was 86%. Table 1 describes the socio-demographic characteristics of our study population (n = 400). Females, people belonging to 29–39 years of age, those who were married, had income > 10,000 – 50,000 Rs. and graduates/postgraduates were in majority.

**Table 1:** Socio demographics of the respondents

Respondents	Frequency (n = 400)	%
**Gender**		

Males	195	48.8

Females	205	51.2

**Mean Age in years**		

Males/Females	43.17/35.43-	

Standard Deviation	±13.489/11.856-	

**Age Distribution (yrs)**		

1.18–28	103	25.8

2.29–39	126	31.5

3.40–50	79	19.7

4.51–61	67	16.8

5.62–72	25	6.2

**Marital status**		

1. Currently single	155	38.8

2. Married	245	61.2

**Income (in rupees)**		

1. <5000	45	11.2

2. 5000–10,000	94	23.5

3. >10,000–50,000	206	51.5

4. >50,000–100,000	43	10.8

5. >100,000	12	3

**Occupation**		

1. Currently Employed	216	54

2. Currently Unemployed	184	46

**Level of education**		

1. Till class 12	145	36.2

2. Graduate/Postgraduate	224	56

3. Can only read and write name	7	1.8

4. Illiterate	6	1.5

5. Diploma	18	4.5

### Knowledge Variables of Gemstone Therapy

As shown in table 2, 252 individuals (63%) in our study population were aware of the use of gemstone therapy for various effects on health. In contrast, only 111 (28%) of the respondents were aware of any method of gemstone use other than wearing a ring or necklace. Of the people who were aware of other methods of using gemstones than rings or necklaces; 51% reported earrings, 26% wrist band and 5% arm bands. With regards to the use of gemstones in treating various ailments, the majority reported that gemstones are helpful in improving gallstones and renal stones; followed by those who held similar beliefs for hypertension, skin diseases and reproductive health. Out of 400 respondents, 46% of the people didn't know if gemstones are helpful in any disease state.

**Table 2:** Knowledge Variables

**Knowledge Variables**	**Frequency**	**%**
**Awareness of use of gemstones for effects on health: (n = 400)**		

Yes	252	63

No	215	31.2

Don't Know	23	5.8

**Awareness of methods of gemstone use other than wearing a ring or necklace: (n = 400)**		

Yes	111	27.8

No	179	44.7

Don't Know	110	27.5

Awareness of the use of gemstones in different diseases (n = 216) *		
		
Gallstones/Renal Stones	111	51.4
Diabetes	47	21.8
Hypertension	1	0.5
Skin	29	13.4
Reproductive Health	25	11.6
Others	3	1.3

* 216 individuals out of 400 were aware of use of gemstones in different disease states. The percentages for this variable have therefore been computed for a total of 216 respondents.

### Belief Variables of Gemstone Therapy

Of the 156(39%) people who expressed a belief that gemstones have an impact on health, 48% believed that gemstones only have a favorable impact on health while 39% believed that gemstone use can exert both favorable and adverse impacts on health. Out of 400 respondents, 34% of the respondents believed that gemstones have an impact on luck only while 21% believed that gemstones can impact both fortune and luck. Seventeen percent people believed that gemstones don't influence either luck or finances. Table 3 provides the remaining details of belief variables regarding gemstone therapy.

**Table 3:** Belief Variables

**Belief Variables**	**Frequency** **(n = 400)**	**%**
**Belief that gemstones have an impact on health**		

Yes	156	39

No	218	54.5

Don't Know	26	6.5

**Belief that gemstones increase physical strength**		

Yes	154	38.5

No	93	23.2

Don't Know	153	38.2

**Opinion that gemstone use is a superstitious belief**		

Yes	247	61.8

No	98	24.5

Don't Know	55	13.8

**Opinion that gemstone use is based on religious beliefs**		

Yes	110	27.5

No	98	24.5

Don't Know	192	48

**Belief that gemstones change colors with one's health**		

Yes	104	26

No	78	19.5

Don't Know	218	54.5

### Advice Variables of Gemstone Therapy

Of the 157 people who had been advised to use a gemstone for a positive impact on health, 33% had been advised by friends, 22% by siblings and 20% by either aunts or uncles. Less than 1% had been advised by their grandparents. Of the 166 respondents who reported that someone among their friends of family members was using gemstones to improve their health, 24% reported friends, 18% siblings, 27% reported aunts or uncles who were currently using gemstones. 42% of the relatives had used ruby/yaqoot/marjan or aqeeq while 11% had used a diamond. 33% had used aquamarine, turquoise or feroza. 55% of the relatives had experienced a favorable impact from the use of gemstones while 31% had experienced both adverse and favorable effects. Out of the 80 respondents who had ever advised anyone to use gemstones for a positive impact on health, 65% had advised friends, 21% parents and 5% had advised siblings. Out of the same 80 individuals, 50% had advised the use of ruby/aqeeq/yaqoot or marjan while 25% had advised the use of sapphire and 11% had advised the use of pearl. Of the 142 who expressed a willingness to advise others about the use of gemstones, 63% said they would advise friends, 18% siblings, and 12% parents. 55% said that they would advise the use of ruby/aqeeq/yaqoot or marjan while 13% said that they would advocate the use of sapphire. Up to 93% people believed that people advised would get a favorable impact on their health secondary to using gemstones while 6% people believed that both adverse and beneficial effects would be the anticipated impact.

#### Practice Variables of Gemstone Therapy

Of the 152 people who reported using a gemstone in the past for a favorable impact on health; 49% had used a ruby/aqeeq/yaqoot or marjan and only 2% reported using an emerald. Wearing a ring was the most popular way of using a gemstone with 84% people reporting its use. 44% of 152 people had used it for a period of 1–5 years while 20% of the people had used gemstones for less than a year. 51% of the people who had used a gemstone reported experiencing a favorable impact on their health, while 40% had experienced both favorable and adverse impacts on their health as a result of using gemstones. Of the 96 people who reported current use of gemstones, 63% reported using a ruby/yaqoot/aqeeq or marjan while 12% reported using sapphire and 7% reported use of pearl. Up to 92% people reported using the gemstone in a ring. Out of the current users of gemstones, 35% had been using it for 1–5 years while 30% had been using it for less than a year. 53% of the current users of gemstones had experienced a favorable impact while 32% had reported experiencing both favorable and adverse impact on health secondary to gemstone use. With regards to the shape of the gemstone, 64% said that their therapeutic gemstone has no specific shape while the majority (22%) reported wearing a spherical gemstone. 72% of the respondents have always worn the same color of the gemstone and out of these, 40% have used red gemstones, 17% blue, 17% brown, and 9% turquoise. 75% people have never considered wearing a combination of different colored gemstones. 54% of the people usually wear a gemstone ring on their ring finger while 27% wear it on the second finger of the hand. Only 26% people believe that gemstones change color with one's state of health while 55% people had never heard of this transition.

**Figure 1:**
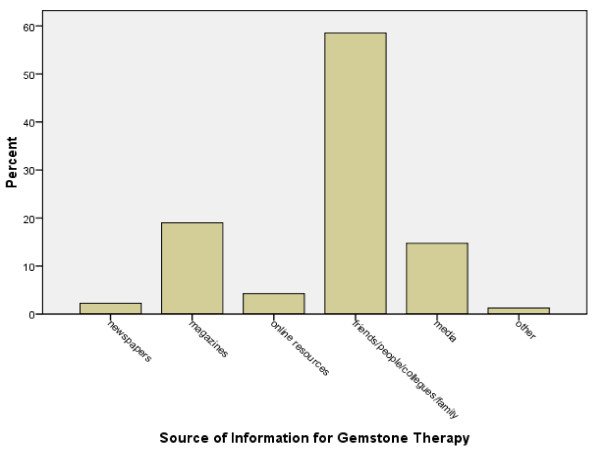
**Source of Information for Gemstone Therapy**.

34% people said that gemstone therapeutics had sometimes worked out the right way for them, while 48% individuals gave a "don't know response". Out of the 40 people who had considered using a cheap substitute for the original gemstones, only 12 (30%) found the substitutes to be as beneficial as the originals. Most of the people had heard about gemstone therapeutics by word of mouth with 59% reporting hearing it from other people, friends, colleagues or family members. Magazines were accredited as a source of information by 19% and other media (such as television and radio) by about 15% of the respondents (Figure 1).

### Chi square/Fisher's Exact Test Results

The belief that gemstones have an impact on health was significantly associated with the use of gemstones (p value = < 0.001), advising others to use gemstones in the future (p value = < 0.001) and duration of time for which gemstones were used (p value = 0.028). It was also significantly associated with age (p value = < 0.001), gender (p value = < 0.001), education (p value = 0.004), occupation (p value = < 0.001) and socioeconomic status (p value < 0.001). [Table 4]

**Table 4:** Associations of belief that gemstones have an impact on health

Variables	**Belief that gemstones have an impact on health**			
	Yes	No	Don't Know	P-values

**Use of Gemstones (n = 400)**				

Yes	98	46	8	< 0.001

No	58	172	18	

**Advising Others to Use Gemstones in Future (n = 400)**				

Yes	68	65	9	< 0.001

No	63	148	16	

Don't Know	156	5	1	

Duration of Gemstone Use (n = 152) *				

- < 1 year	25	3	3	

- 1 – 5 years	38	26	3	0.028

- 5 – 10 years	18	14	1	

- > 10 years	17	3	1	

**Gender (n = 400)**				

Male	82	111	2	< 0.001

Female	74	107	24	

**Socioeconomic Status (n = 400)**				

< 5,000 Rs	12	30	3	< 0.001

5 – 10,000 Rs	34	49	11	

10 – 50,000 Rs	74 120	12		

> 50, 0000 Rs	36	19	0	

**Education (n = 400)**				

Can only read and write name	11	0	2	

Upto Class 8	7	15	0	0.004

Upto Class 12	42	73	8	

Graduate/Post graduate/Diploma	96	130	16	

**Age in years (n = 400)**				

18 – 28	17	74	12	< 0.001

29 – 39	52	64	10	

40 – 50	41	36	2	

51 – 61	36	29	2	

62 – 72	10	15	0	

**Occupation (n = 400)**				

Student	1	37	3	

Self-employed	26	43	3	

Private Service	36	51	3	

Government Service	19	27	3	< 0.001

Unemployed	0	2	4	

Retired	15	12	0	

Housewife	57	43	10	

Others	2	3	0	

* 152 individuals out of 400 had used gemstones in the past for effects on health. Therefore, association for duration of usage is reported for these 152 people.

Receiving advice about gemstone use in the past was significantly associated with advising others of gemstone use in the future (p value = 0.020 at 95% CI). Gender, education, occupation and socioeconomic status were significantly associated with the opinion that gemstone use for health improvement is based on a superstitious belief (Gender: p value = 0.002, Education: p value = < 0.001, Occupation: p value = 0.023 and Socioeconomic status: p value = 0.002). [Table 5] Age, education, occupation and socioeconomic status were significantly associated with the opinion that use of gemstones for health improvement is based on religious beliefs (Age: p value = 0.020, Education: p value = < 0.001, Occupation: p value = < 0.001 and SES: p value = 0.008). [Table 6]

**Table 5:** Association that gemstone use is based on superstitious beliefs

**Variables**	**Opinion that gemstone use for health improvement is based on a superstitious belief (n = 400)**			
	**Yes**	**No**	**Don't Know**	**p-values**

**Gender**				

Male	113	62	20	0.002

Female	134	36	35	

**Socioeconomic Status**				

< 5,000 Rs	35	6	4	0.002

5 – 10,000 Rs	49	35	10	

10 – 50,000 Rs	124	52	30	

> 50, 0000 Rs	39	5	11	

**Education**				

Can only read and write name	7	2	4	

Upto Class 8	6	16	0	< 0.001

Upto Class 12	61	50	12	

Graduate/Post graduate/Diploma	173	30	39	

**Occupation**				

Student	32	3	6	

Self-employed	43	26	3	0.023

Private Service	48	24	18	

Government Service	33	10	6	

Unemployed	5	1	0	

Retired	20	5	2	

Housewife	62	28	20	

Others	4	1	0	

**Table 6:** Association of gemstone use based on religious beliefs

Variables	**Opinion that gemstone use for health improvement is based on religious belief (n = 400)**			
	Yes	No	Don't Know	**p-values**

**Age in years**				

18 – 28	25	31	47	0.02

29 – 39	29	30	67	

40 – 50	25	9	45	

51 – 61	20	21	26	

62 – 72	11	7	7	

**Socioeconomic Status**				

< 5,000 Rs	13	11	21	0.008

5 – 10,000 Rs	16	20	58	

10 – 50,000 Rs	59	60	87	

> 50, 0000 Rs	22	7	26	

**Education**				

Can only read and write name	4	3	6	

Upto Class 8	0	0	22	< 0.001

Upto Class 12	36	29	58	

Graduate/Post graduate/Diploma	70	66	106	

**Occupation**				

Student	5	17	19	

Self-employed	12	16	44	< 0.001

Private Service	22	22	46	

Government Service	8	10	31	

Unemployed	4	1	1	

Retired	16	6	5	

Housewife	40	26	44	

Others	3	0	2	

Use of gemstones for effects on health was significantly associated with age (p value = < 0.001), marital status (p value = < 0.001), occupation (p value = < 0.001) and use of gemstones by friends or family members (p value = < 0.001). [Table 7]

**Table 7:** Association of use of gemstones with effects on health

Variables	**Use of gemstones for impact on health (n = 400)**		
	Yes	No	**p-values**

**Age in years**			

18 – 28	18	85	< 0.001

29 – 39	49	77	

40 – 50	35	44	

51 – 61	39	28	

62 – 72	11	14	

**Occupation Use**			

Student	5	36	

Self-employed	25	47	< 0.001

Private Service	32	58	

Government Service	21	28	

Unemployed	0	6	

Retired	17	10	

Housewife	50	60	

Others	2	3	

**Marital Status**			

Married	111	134	

Single	34	106 < 0.001	

Divorced	1	0	

Widowed	6	8	

**Friends or Family Using Gemstones to Improve Health**			

Yes	83	83	

No	60	133 < 0.001	

Don't Know	9	32	

Awareness of the use of gemstones for health was not significantly associated with the belief that gemstones have an impact on health (p value = 0.580) or past use of a gemstone (p value = 0.228). Age (p value = 0.001), education (p value = < 0.001), and income (p value = 0.002) were, however, all significantly associated with the awareness that gemstones are used for their effects on health. [Table 8]

**Table 8:** Awareness of the use of gemstones for their effects on health

Variables	**Awareness of the use of gemstones for their effects on health (n = 400)**			
	Yes	No	Don't Know	**p-values**

**Age in years**				

18 – 28	65	36	2	0.001

29 – 39	80	31	15	

40 – 50	59	18	2	

51 – 61	33	32	2	

62 – 72	15	8	2	

**Socioeconomic Status**				

< 5,000 Rs	30	15	0	0.002

5 – 10,000 Rs	48	38	8	

10 – 50,000 Rs	127	66	13	

> 50, 0000 Rs	47	6	2	

**Education**				

Can only read and write name	1	8	4	

Upto Class 8	17	5	0	< 0.001

Upto Class 12	70	47	6	

Graduate/Post graduate/Diploma	164	65	13	

### Multiple Logistic Regression

The following variables were subjected to the multiple regression analysis: 'age', 'education', 'occupation' and 'income'. Table 9 shows the independent predictors of awareness of use of gemstones for their impact on health. The significant (*p *< 0.05) independent predictors of awareness of use of gemstone therapy included: age > 39 years, education > grade 5, income > Rs.50,000.

**Table 9:** Multiple logistic regression for awareness of use of gemstones

Variables	Awareness n = 252 (%) $	Adjusted OR *	95% CI **
**Age in years (p = 0.001)**			

18–28	65 (25.8)	1	

29–39	80 (31.8)	1	0.6 – 1.7

40–50	59 (23.4)	1.7	0.9 – 3.3

51–61	33 (13.1)	0.6	0.3 – 1.1

62–72	15 (5.9)	0.9	0.4 – 2.2

**Education (p = 0.000)**			

Up to grade 5	13 (5.2)	1	

Up to grade 8	5 (1.9)	1.8	0.4 – 9.1

Up to grade 12	70 (27.8)	1.4	0.6 – 3.3

Beyond grade 12	164 (65.1)	2.3	1.0 – 5.1

**Occupation (p = 0.093)**			

Currently unemployed	117 (46.4)	1	

Currently employed	135 (53.6)	1.1	0.6 – 1.8

**Monthly income in rupees (p = 0.004)**			

< 5,000	30 (11.9)	1	

5,000 – 10,000	48 (19)	0.5	0.3 – 1.1

10,000 – 50,000	127 (50.4)	0.8	0.4 – 1.6

> 50,000	47 (18.7)	3	1.1 – 7.8

* OR = Odds Ratio			

** CI = Confidence Interval

$ 252 out of 400 people were aware of use of gemstones for effects on health.

Table 10 shows the independent predictors of belief that gemstones use has an impact on health. Following were significant (*p *< 0.05) independent predictors of the belief that gemstones have an impact on health: male gender, age > 28 years, married, education up to grade 5, currently employed and income > Rs. 5,000.

**Table 10:** Multiple logistic regression for belief that gemstones impact health

**Variables**	**Belief (n = 156)** **n (%)^$^**	Adjusted OR*	95% CI **
**Gender (p = 0.000) **			

Male	82 (52.7)	1	

Female	74 (47.3)	0.8	0.5 – 1.2

**Age in years (p = 0.000)**			

18-28	17 (10.9)	1	

29-39	52 (33.3)	3.6	1.9 – 6.8

40-50	41 (26.3)	5.5	2.8 – 10.8

51-61	36 (23.1)	5.9	2.9 – 11.9

62-72	10 (6.4)	3.4	1.3 – 8.8

**Marital Status (p = 0.000)**			

Divorced/Widowed/Separated	5 (3.2)	1	

Married	120 (76.9)	1.9	0.6 – 5.8

Single†	31 (19.9)	0.6	0.2 – 1.8

**Education (p = 0.004)**			

Up to grade 5	14 (8.9)	1	

Up to grade 8	4 (2.7)	0.9	0.2 – 4.5

Up to grade 12	42 (26.9)	0.5	0.2 – 1.1

Beyond grade 12	96 (61.5)	0.6	0.3 – 1.4

**Occupation (p = 0.000) **			

Currently unemployed	73 (46.8)	1	

Currently employed	83 (53.2)	2.3	1.2 – 4.2

**Monthly income in rupees (p = 0.001)**			

< 5,000	12 (7.7)	1	

5,000 – 10,000	34 (21.8)	1.6	0.7 – 3.4

10,000 – 50,000	74 (47.4)	1.5	0.7 – 3.2

> 50,000	36 (23.1)	5.2	2.2 – 12.4

* OR = Odds Ratio			

** CI = Confidence Interval			

† These were the respondents who never married

$ 156 individuals had a belief that gemstones impact health

## Discussion

Correlations of socio-demographic variables with awareness, use, and belief towards gemstones demonstrate interesting findings. A higher prevalence of the awareness about the use of gemstones and their effects on health exists in individuals who are educated; with 89 out of 252 such respondents being graduates, and belonging to the middle class stratum of the society, with an income ranging from Rs. 10,000–50,000 (1 US $ equivalent to 60.667 Pak rupee(PKR)). We also found a significant association between age and an awareness of the use of gemstones. The young population (29–39 years of age) had the highest prevalence of awareness regarding the use of gemstones. The same age group also formed the majority of those who had used a specific gemstone, with 49% of them having used one of these gemstones – ruby, yaqoot, marjan, or aqeeq, as a ring (91%). It was noted that 61% of those who believed in the healing power of gem therapy stated that using gemstones tends to have a favorable impact on health and mostly from those who had used a particular one. The belief that gemstones have an impact on health was also found to be significantly associated with age, gender, education, occupation, marital status, and socioeconomic status (SES) of the respondents.

Other important implications can be derived from the significant associations we found between advice and use of gemstones. Of the 39% of those who had used gemstones or 38% of those who held any beliefs regarding their use, around one-third (36%) stated that they would advise others about use of gemstones, mostly their friends (63%). It was observed that 49% of those who had been advised about the use of gemstones showed an inclination towards their future use. An interesting association between marital status and level of education was observed with the desire to advise others to use gemstones for their effects on health. Married and educated people showed greater inclination to advise someone in the future about the use of gemstones.

The majority of the people reported that gemstones are helpful in improving gallstones, renal stones, hypertension, skin diseases, and reproductive health. A literature search revealed that gemstone therapy is believed to have beneficial effects on the course of chronic diseases, such as migraine, asthma, tumors, backaches, hypertension, kidney diseases and stones, piles, and arthritis [4].

In our survey, different people reported wearing different stones. This difference in practices is explainable by the fact that different stones are believed to be helpful in different disease states. Wearing a ring emerged as the most popular way of using a gemstone. Gemstones are sported in different ways. One source reported that the best way for the increased efficiency of gemstone therapy is to sport them at the fingers. The fingers are believed to have direct channels to the centers of the human body. For exact balancing of the negative and positive energies of centers, different kinds of gems may be worn [4].

Those who showed willingness to use a therapeutic gemstone in the future mentioned that the use of or advice by family members was the main reason for their willingness. When inquired about anyone they know who uses a gemstone, 41% of these respondents reported the use of a gemstone by a friend or a family member. This constitutes a type of "lay referral system," whereby people are inclined to try out a treatment based on word of mouth from an acquaintance. In another study, the most common reason for using CAM was being taught to do so by family members [8]. Opting for CAM use stems from an intricate interaction of an array of dynamic factors that reflect variations in sociocultural factors influencing reasoning, illness comprehension, treatment-seeking behaviors, motivations, attitudes, and availability. Factors, such as residence in rural areas, acceptability of service, cooperation, empathetic attitude and active listening by the traditional healers as compared to conventional health care professionals, [21,24] also play a part. In Pakistan, the joint family system is prevalent; elders pass down their life experiences as part of a legacy to their children and advise them about what they have found beneficial in their lives. Besides family advice and pressure, other reasons for consulting a CAM healer could be the proximity, affordable fee, availability of the provider, and the strong opinion of the community [21].

Almost an equal number of respondents believed that gemstone therapy can have a role in improving one's health as well as physical strength. Out of these, a significant majority did not know if gemstones changed colour with one's state of health, reflecting that people in this part of the world are yet not aware of the scientific basis of gemstone/colour therapy; the majority having used a specific gemstone (specially red/brown stone) because it was either used or advised by someone by a friend or family member, reinforcing the aforementioned statement regarding the significance of 'lay referral' system in this country. Therefore we feel that there is a need to educate people regarding the actual basis of therapeutic benefits of gemstone therapy and how this energy medicine offers solutions to seemingly intractable health problems.

An almost bi-modal trend was observed regarding the effects of gemstones; respondents reported favorable effects as well as 'both favorable and adverse' effects experienced from using the gemstones, (78% and 61%, respectively). This is consistent with different beliefs regarding gemstone therapy; some stones are believed to channel positive energy while others are believed to have adverse effects as mentioned earlier. Except for the 4% of the respondents who reported that gemstones had never worked out the right way for them, the majority expressed satisfaction with gemstone therapy.

Categorizing the use of gemstones as being based on either superstitious or religious beliefs emerged as a controversial issue. 62% of the respondents agreed that the use of gemstones is based on superstitious beliefs. However, only about 28% of the respondents stated religious beliefs as a basis for gemstone use. The belief that use of gemstones is based on superstition had a strong association with gender, SES, occupation and education of the respondents. On the other hand, the opinion that use of gemstones is based on religious beliefs was significantly associated with education, occupation and SES.

## Strengths and limitations

To the best of our knowledge, this study is the first of its kind and provides a valuable insight into the reasons motivating people to use gemstones. Additionally, our questionnaire on assessing knowledge, attitudes and practices of gemstone therapeutics may be employed in future studies as a template for gauging similar parameters.

We also acknowledge the following limitations of our study. Firstly, we used convenience sampling to draw our sample. Convenience sampling is inferior to probability sampling in its representativeness of the population, and this limits the external validity of the study. Sampling from a single centre limits the generalizeabilty of the study. However, as mentioned before, CHC receives people from all socioeconomic classes, helping offset this limitation. Our findings are generalizable to a wider population because people in Pakistan simultaneously use different delivery systems of its pluralistic health system. Health seeking behavior is shaped by the type of symptoms experienced and the number of days of illness. Milder symptoms like fever are managed with home remedies or folk prescriptions, while more complex, prolonged or severe symptomatology is usually managed in consultation with biomedical health provider [21]. The respondents in the study had used gemstones either currently or previously and they were encountered while seeking medical advice from a medical doctor at CHC at AKUH, Karachi, Pakistan.

Hospital attendees were chosen for convenience of administration of interviews. We acknowledge that the knowledge, attitudes and practices of individuals presenting to the hospital may be different than those encountered in the community setting.

The information in the survey was acquired via a face-to-face interview which was based on a questionnaire. While this may have led to higher rates of completion of the forms because of interviewer's encouragement for optimum completion, it may also have introduced interviewer's bias in the process of data collection despite all efforts to minimize it.

We did not ascertain the exact reason for which the respondents were attending the CHC clinics. Future studies can be undertaken to focus on this particular aspect to explore any correlations between the nature of health problem that brought them to the clinic and the use of gemstones. Also, the exact nature of the use of gemstone (cosmesis and aesthetics versus medical ailments) can be ascertained in greater detail in future studies. In addition, future studies can be done to explore the simultaneous use and associations, if any, of gemstone therapy and other modalities of CAM and how they may correlate to each other.

## Conclusion

This study provides an insight into the knowledge, beliefs and practices with regards to use of gemstone therapy among the population. More than one third of our study population has used gemstones in the past while more than one fifth is currently using gemstones. More than half of our sample population is aware of the use of the gemstones for their various effects. Willingness to use gemstones is associated with the beliefs about the impact of gemstone therapy on health. Friends and family seem to be the major role players influencing people's willingness to use gemstones. Superstition and to a lesser extent, religion continue to have an impact on people's way of thinking with regards to gemstone therapy.

Today, alternative tools for transforming health are being embraced by a larger number of people, and many countries now have pluralistic health care systems. CAM modalities have excited the interest of physicians and medical researchers alike. In a time when we are all looking for a cure and realizing that many of the traditional treatment methods are not only laden with side effects, but simply may not work as well as expected, gemstone therapy certainly warrants scientific inquiry.

We should acknowledge that CAM is an art mushrooming throughout the world and many people regard it as a panacea for their problems. Ignorance on the part of conventional medical practitioners about these practices poses the serious risk of alienating the public [12]. Based on our findings, we propose gathering validated information with regards to a multifaceted phenomenon such as gemstone therapy – this might open new vistas in the field of medicine and benefit a larger number of patients.

## Abbreviations

AKUH: Aga Khan University and Hospital; CHC: Community Health Centre; SES: Socioeconomic Status

## Competing interests

The authors declare that they have no competing interests.

## Authors' contributions

**SI **was involved in study conception, data collection, entry, analysis, revision and manuscript writing. **TS **was involved in study conception, data collection, entry, analysis, revision and manuscript writing. **WQ **was involved in study conception, manuscript writing, revision, editing and overall supervision. All authors have read and approved the final manuscript.

## Pre-publication history

The pre-publication history for this paper can be accessed here:



## Supplementary Material

Additional file 1 **Questionnaire of Survey**. This is the questionnaire used for the survey to gauge the knowledge, attitudes and practices of the selected adult population about gemstone therapeutics. Click here for file
