# Supplementary material for: Knowledge, attitudes and practices regarding gemstone therapeutics in a selected adult population in Pakistan
Source: BMC Complement Altern Med. 2009 Aug 26;9:32. doi: 10.1186/1472-6882-9-32 (PMC2739841; doi:10.1186/1472-6882-9-32)
Supplement: Additional file 1 — Questionnaire of Survey. This is the questionnaire used for the survey to gauge the knowledge, attitudes and practices of the selected adult population about gemstone therapeutics. [file 1472-6882-9-32-S1.doc]

**QUESTIONNAIRE FOR GEMSTONE STUDY**

**This is a questionnaire that aims to assess the knowledge, attitudes and practices about gemstone therapy. Kindly spare some moments of your precious time and help us in our research by sharing your experiences with us. Any suggestions are most welcome!**

**Demographics**

1.1 Age:- ______________ in years

1.2 Sex:- ⬜ M ⬜ F

1.3 Marital status:-

⬜ (a) Married

⬜ (b) Single

⬜ (c) Widow

⬜ (d) Separated

⬜ (e) Divorced

1.4 Education:-

⬜ (a) Illiterate

⬜ (b) Can read and write

⬜ (c) Primary(upto class V)

⬜ (d) Secondary (upto class VIII)

⬜ (e) Matric/Cambridge (upto class X)

⬜ (f) Intermediate/A’Levels(upto class XII)

⬜ (g) Graduate

⬜ (h) Post graduate

⬜ (i) Diploma

1.5 Occupation:-

⬜ (a) Student

⬜ (b) Self employed

⬜ (c) Private service

⬜ (d)Government employee

⬜ (e)Unemployed

⬜ (f)Retired

⬜ (g)Housewife

⬜ (h)Any other

1.6 Income:-

(a) <5000 a month ⬜

(b) 5000-10,000 a month ⬜

(c) 10,000-50,000 a month ⬜

(d) 50,000-100,000 a month ⬜

(e) >100,000 a month ⬜

**2.1- Are you aware that gemstones are used for their affects on health?**

(a)- Yes ⬜

(b)-No ⬜

(c) Don’t know ⬜

**2.2 - Do you believe that gemstones have on impact on health?**

(a)- Yes ⬜

(b)-No ⬜

(c) Don’t know ⬜

**2.2 A-If “Yes” then,**

(a)-adverse impact ⬜

(b)-favourable impact ⬜

(c)-Both. ⬜

**2.3-Have you ever used a gemstone to have a favourable impact on your health?**

(a)- Yes ⬜

(b)-No ⬜

# 2.3A- If “Yes then, state

(a)-Gemstone used:-…………………..

(b)-How used? …………………………

(c)-For how long?

1)- <1 year 2)- 1-5 years 3)-5-10 years 4)->10 years 5)- since childhood

(d)- Its impact on your health:-…………………………………………………………….

# 2.4-Do you presently use any gemstone to have a favorable impact on your health?

(a)- Yes

(b)-No

If “Yes” then, state

(a)-gemstone used:-…………………..

(b)-How used? ……………………….

(c)-For how long?

1)- <1 year 2)- 1-5 years 3)-5-10 years 4)->10 years 5)- since childhood

(d)- Its impact on your health:-…………………………………………………………….

2.5-Have you ever been advised to use a gemstone to have positive impact on your health?

(a)- Yes

(b)-No

If “Yes” then, state

(a)-By whom?..................................................................

(b)-What gemstone?..........................................................

(c)- How used if used?.......................................................

(d)-Impact, if any:-………………………………………..

# 2.6-Does anyone in among your friends or family use gemstone to improve health?

(a)- Yes

(b)-No

(c) Don’t know

If “Yes” then, state

(a)-Who?.................................................

(b)-Which gemstone?...............................

(c)-Impact:-………………………………

# 2.7- Will you use gemstone if advised?

(a)- Yes ⬜

(b)-No ⬜

(c) Don’t know ⬜

2.8- Have you ever advised anyone to use gemstone to have positive impact on health?

(a)- Yes

(b)-No

If “Yes” then, state

(a)-Who?.................................................

(b)-Which gemstone?...............................

(c)-Impact:-………………………………

# 2.9- Will you advise anyone to use questions for beneficial impact on health?

(a)- Yes

(b)-No

If “Yes” then, state

(a)-Who?.................................................

(b)-Which gemstone?...............................

(c)- Anticipated impact:-………………………………

# 2.10-Do you believe that use of gemstones has an impact on

(A)-Luck

(a)- Yes (b)-No (c) Don’t know

(B)-Finance

(a) Yes b)-No (c) Don’t know

(C)- Longevity

(a) Yes b)-No (c) Don’t know

Any other……………………………………………………..

**2.11- Please state gemstones helpful in different diseases in your opinion:-**

(a)- Gall stones…………………………

(b)- Renal stones……………………….

(c)- Others……………………………..

2.12- Do you know of any other method of use of gemstones for beneficial impact on health other than wearing a ring or a necklace?

(a)- Yes

(b)-No

(c) Don’t know

If “Yes” then state the method………………………………………..

# 2.13-Do you agree that wearing certain gemstones increases physical strength?

(a)- Yes

(b)-No

(c) Don’t know

2.14-Do you agree that use of gemstones for health improvement is a superstitious belief?

(a)- Yes ⬜

(b)-No ⬜

(c) Don’t know ⬜

# 2.15-Do you agree that use of gemstones for health is based on religious beliefs?

(a)- Yes ⬜

(b)-No ⬜

(c) Don’t know ⬜

# 2.16- Do you wear your birthstone as a therapeutic gemstone?

(a)- Yes ⬜

(b)-No ⬜

(c)-I don’t know what my birthstone is. ⬜

# 2.17:- Is their a specific shape of a therapeutic gemstone that you wear?

1. Sphere
2. Square
3. Triangle
4. Rhombus
5. Any other shape

**2.18:- Do you always wear the same color of gemstone? If yes then state the color:-**………………………………

# 2.19:- Which accessory do you prefer to wear?

1. Ring
2. Necklace
3. Armband
4. Wristband
5. Earing
6. Any other

2.20:- Have you ever considered wearing a combination of different colored gemstones?

1. Yes , (a combination of two)
2. Yes , (a combination of three)
3. Yes , (a combination of more than three)
4. Never

# 2.21:- On which finger do you usually wear gemstone ring?

1. First
2. Second
3. Third
4. Pinky
5. Thumb

**2.22:- Do you believe that a gemstone changes color with one’s state of health?**

1. Yes
2. No
3. Don’t know/never heard of

**2.23-Do the gemstone therapeutics always work out the right way for you?**

(a) Yes, most of the time ⬜

(b) Often ⬜

(c) Sometimes ⬜

(d)- Never ⬜

2.24-Have you ever considered wearing cheaper substitutes for the original and precious gemstones?(eg, a red stone for ruby)

(a)- Yes ⬜

(b)- No ⬜

**2.25- Do the substitutes prove as beneficial as the original?**

(a)- Yes ⬜

(b)-No ⬜

(c)-Don’t know. ⬜

**2.26- How did you come to know about gemstone therapeutics?**

(a) Newspapers ⬜

(b) Magazines ⬜

(c) Online resources⬜

(d) Friends/colleagues/family. ⬜

(e) Media ⬜

(f) Any other⬜
